# A population-based study of severe, less common comorbidities in Duchenne muscular dystrophy

**DOI:** 10.1007/s00415-025-13323-6

**Published:** 2025-08-28

**Authors:** Lisa Wahlgren, Sara Nordström, Már Tulinius, Anna-Karin Kroksmark, Kalliopi Sofou

**Affiliations:** 1 Department of Pediatrics, Institute of Clinical Sciences, University of Gothenburg, Gothenburg, Sweden; 2 The Queen Silvia Children’s Hospital, Sahlgrenska University Hospital, Member of the European Reference Network EURO-NMD, Gothenburg, Sweden; 3 Department of Neurology, Neuromuscular Centre, Sahlgrenska University Hospital, Gothenburg, Sweden; 4 Department for Health and Rehabilitation/Physiotherapy, University of Gothenburg, Gothenburg, Sweden

**Keywords:** Duchenne muscular dystrophy, Comorbidities, Complications, Incidence, Disease outcome, Rare

## Abstract

**Background:**

Since the increasing longevity and the advent of new therapeutic modalities in Duchenne muscular dystrophy (DMD), comprehensive clinical surveillance is of paramount importance. Our study aimed to examine the occurrence of severe, less common comorbidities among patients with DMD and their impact on life expectancy and overall disease burden.

**Methods:**

This was a retrospective, nationwide study of all male patients with DMD who were followed at a medical clinic in Sweden, born and deceased during the period 1970–2019. Data regarding cause-of-death and comorbidities were retrieved by the medical records and the Cause of death Registry. The assessed variables were defined as ‘severe’ when depicting comorbidities with potentially serious, life-threatening outcomes.

**Results:**

Of the 129 included patients, approximately 56% presented with at least one severe complication or event, the most common being gastrointestinal complications and life-threatening arrhythmias, with incidence rates of 56.8 and 48.3/10,000 person-years, respectively. Acute kidney injury with an incidence of 16.6/10,000 person-years, was primarily seen within the months preceding death. Vascular events occurred in 10.7% of the patients, mainly cerebrovascular events and venous thromboembolism -including fatal post-traumatic pulmonary embolism-, each with an incidence of 23.5/10,000 person-years. Fracture occurrence, predominantly seen among non-ambulatory patients, had an incidence of 310/10,000 person-years. We further studied cumulative incidences and associations with loss of ambulation and glucocorticoids.

**Conclusion:**

Our study provides new insights into severe comorbidities in DMD, emphasizing the need for lifelong disease monitoring, especially considering that many complications are subject to prevention and, if detected early, to successful treatment.

**Supplementary Information:**

The online version contains supplementary material available at 10.1007/s00415-025-13323-6.

## Introduction

Duchenne Muscular Dystrophy (DMD) is the most common form of muscular dystrophy in childhood and adolescence, affecting approximately 7 per 100.000 males [1]. It is caused by mutations in the Xp21.1-p21.2 DMD gene that encodes the 427-kDa cytoskeletal protein dystrophin. The loss of functional dystrophin in the muscle fibers leads to gradual muscle weakness, loss of independent ambulation by adolescence and progressive decline of respiratory and cardiac function. The life expectancy is currently estimated between 24 and 30 years, with cardiopulmonary failure being the leading cause of death [2, 3].

The clinical phenotype of DMD is complex and in major part dependent on the progressive muscle degeneration and the cardiopulmonary complications that follow. Besides the 79 exons, the DMD gene also consists of 7 internal promoters expressing different dystrophin isoforms in different tissues other than the skeletal muscles, such as the central nervous system, cardiac muscle, lungs, kidneys, liver and spleen. Some tissues such as the skeletal muscles completely lack dystrophin in DMD, while other tissues show variable expression of dystrophin. This partly explains the clinical complexity of DMD and the varying degree of comorbidities that occur, such as gut dysmotility, intellectual disability, neurodevelopmental and psychiatric symptoms [4]. The use of high-dose glucocorticoids as per the standards-of-care is another significant contributor to clinical heterogeneity, being associated not only with positive clinical outcomes but also with side effects, such as obesity, osteoporosis and cataract [5].

After more than two decades of merely using glucocorticoids and symptomatic treatment, we are now witnessing the emergence of novel therapies in the field of DMD. The adeno-associated virus (AAV)- mediated microdystrophin is a gene therapy that was recently approved by the FDA for the treatment of pediatric patients with DMD [6, 7]. Other therapies include antisense oligonucleotides (ASO)-mediated exon skipping and small molecules targeting specific mutations of the dystrophin gene [8]. This accelerating innovation in therapeutic interventions is anticipated to fundamentally alter the natural history of DMD. Hence, characterizing the evolving phenotype has never been more essential.

To live with a chronic debilitating disease such as DMD is challenging not only due to the major motor impairment and severe cardiopulmonary disease. There are other, less prevalent comorbidities following the disease progression that often go undetected and untreated until they cause severe complications. Our study aimed to examine the occurrence of severe, less common comorbidities among patients with DMD and their impact on life expectancy and overall disease burden.

## Methods

### Study design

This was a retrospective, population-based, nationwide study of all male patients living in Sweden, born and deceased during the period 1970–2019. We included all patients with a consistent clinical phenotype of DMD with increased serum creatine kinase and at least one of the following: (i) typical muscle biopsy findings; (ii) pathogenic DMD variants in the dystrophin gene; (iii) a confirmed DMD diagnosis in a maternal relative. The clinical phenotype was defined as symptom onset before 5 years of age and either loss of ambulation before 13 years of age, or 16 years of age for patients receiving glucocorticoids. Only patients who were followed at a medical clinic in Sweden were included. The overall study design, life expectancy and causes of death have been presented in detail elsewhere [3, 9].

### Definition of variables

As this was a retrospective medical record review, a standardized case report form was used for data collection. We then performed a quality assessment and if data for a specific variable were incomplete, the patient was not classified as an 'observed patient' for that variable. The assessed variables were grouped and defined as follows:

A. Gastrointestinal (GI) dysfunction and complications

Dysphagia was defined as swallowing difficulties requiring nasogastric tube feeding or gastrostomy. Upper GI complications included esophagitis, gastritis, peptic ulcer and/or duodenal disease. Motility disorder included dyspepsia, emesis, regurgitation, abdominal distension, gastroparesis, chronic constipation and/or alternating diarrhea/dyschezia.

Any of the following were considered severe GI complications: GI bleeding, volvulus, ileus, intestinal pseudo-obstruction or life-threatening GI infection.

B. Vascular events

Any of the following were considered: vasculitis/vasculopathy, any cerebrovascular event and venous thromboembolism (i.e. pulmonary embolism and deep venous thrombosis). Coronary embolism was not included in this variable.

C. Renal complications

Any of the following were considered: recurrent urinary tract infections, bladder dysfunction (i.e. urinary incontinence, retention/hydronephrosis), acute kidney injury and chronic renal failure. Nephrolithiasis, isolated hematuria and single urinary tract infections were not included in this variable.

D. Acute cardiac events

Any of the following were considered: myocardial infarction and/or arrhythmia requiring cardioversion or clearly associated to death. Being a cardinal feature of the disease phenotype, heart failure was not considered as an acute cardiac event.

The term ‘severe’ was used to characterize comorbidities with potentially serious, life-threatening outcomes. In addition to the severe GI complications, renal complications, vascular and acute cardiac events summarized above, severe comorbidities also included hepatic, biliary or pancreatic disorder, as well as sepsis. Cardiopulmonary conditions characteristic of the disease phenotype were not considered comorbidities, except for acute cardiac events.

### Data analysis

Descriptive statistics were presented as medians with interquartile ranges (IQRs) for continuous variables, and as counts and percentages for categorical variables. Group comparisons for categorical variables were performed using the Pearson chi-square and the Fisher’s exact tests. Incidence rates were calculated as number of events/total person-years of observation. Cumulative incidence functions were used to describe the cumulative incidence of events over time, accounting for competing events. As collected data covered nearly five decades, we also performed stratified analysis for two birth cohorts, i.e. patients born 1970–1989 and 1990–2009, to better assess differences in event occurrence over time. There were no patients born since 2010 that had deceased by 2019, therefore this birth cohort was not included in the analysis. Associations between severe complications or events and causes of death were analyzed using cause- specific Cox proportional hazards regression with censoring for competing events. All significance tests were two-sided and conducted at the 5% significance level. Analyses were performed using IBM SPSS 30.0, SAS version 9.4 (SAS Institute Inc., Cary, NC, USA) and R version 4.4.1 (R Core Team, Vienna, Austria).

## Results

**Fig. 1:**
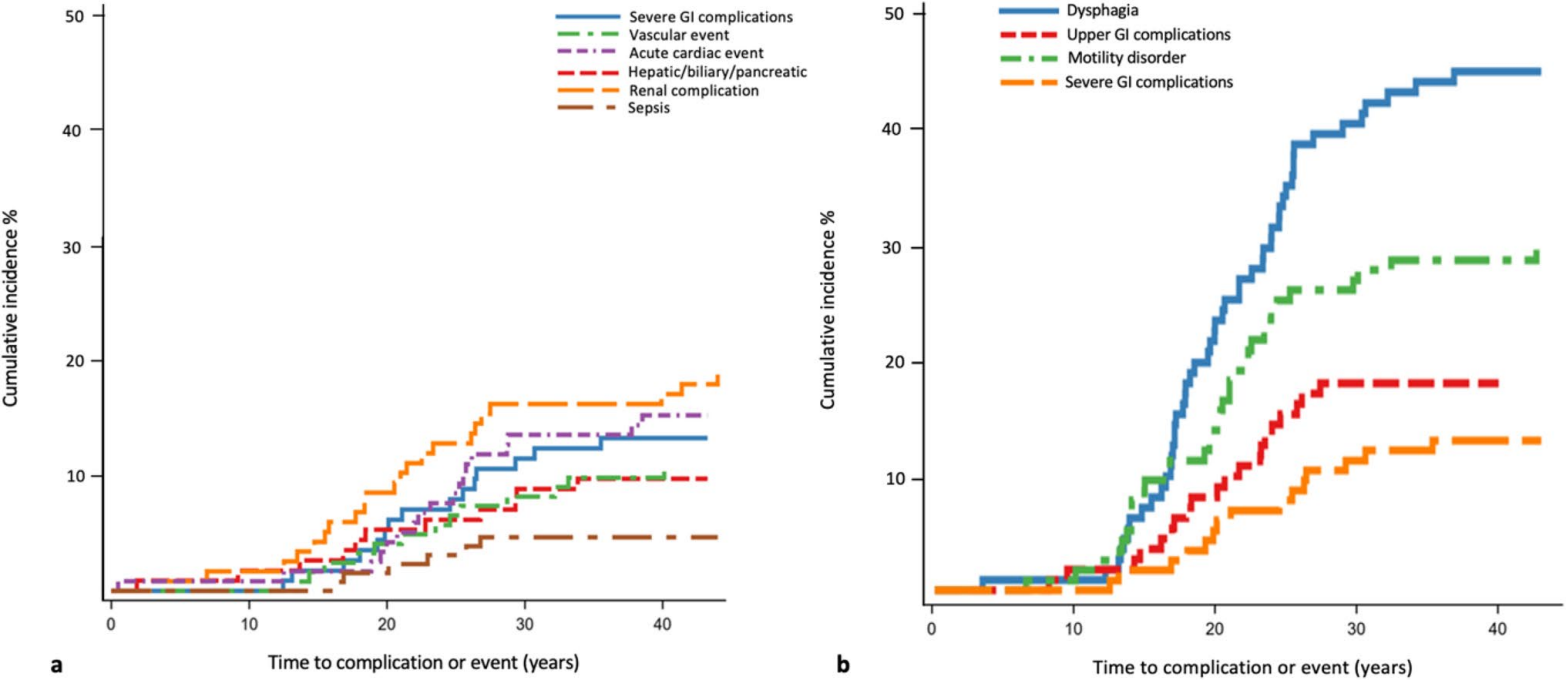
**a**, **b** Cumulative incidence for **a** severe complications and events; **b** gastrointestinal (GI) dysfunction and severe GI complications

Of the 129 male patients that were identified and included in the study, 20% died of non-cardiopulmonary causes at a median age of 19.5 years old (IQR 16.2–25.3, n = 26). The median age of death for the remaining patients was 24.8 years old (IQR 20.4–29.7, n = 103). Forty-five patients (35%) had genetically confirmed diagnosis based on any of the following: deletion (73.3%), duplication (8.9%), both deletion and duplication (4.4%) and nonsense mutation (13.3%). Approximately 60% of patients had received glucocorticoids, half of whom had discontinued the treatment after a median of 11 years old (IQR 10.3–14.6, n = 31). At least one severe complication or event was seen in 56.2% of patients (n = 68/121), the most prevalent being severe renal complications (n = 28), followed by acute cardiac events (n = 19) and severe GI complications (n = 16). The cumulative incidence of severe complications and events is depicted in Fig. 1a and detailed in Table 1 along with the event occurrence per birth cohort.

**Table 1:** Event rate, cumulative incidence and number of events per birth cohort for (a) severe complications and events, (b) gastrointestinal dysfunction and (c) fractures

	Event rate (%)	Cumulative incidence (95% CI)	Events per birth cohort 1970–1989; 1990–2009
a. Severe complications or events			
Renal complications	22/114 (19.3)	0.19 (0.12, 0.27)	15; 7 (p = 1.000)
Acute cardiac event	19/117 (16.2)	0.16 (0.09, 0.23)	15; 4 (NA)
Severe GI complications	15/113 (13.3)	0.13 (0.08, 0.20)	11; 4 (NA)
Vascular event	13/122 (10.7)	0.11 (0.06, 0.17)	9; 4 (NA)
Hepatic/biliary/pancreatic	11/113 (9.7)	0.10 (0.05, 0.16)	7; 4 (NA)
Sepsis	6/129 (4.7)	0.05 (0.02, 0.09)	4; 2 (NA)
b. GI dysfunction			
Dysphagia	50/109 (45.9)	0.46 (0.36, 0.55)	41; 9 (p = 0.004)
Motility disorder	34/115 (29.6)	0.30 (0.22, 0.39)	27; 7 (p = 0.148)
Upper GI complications	20/109 (18.3)	0.18 (0.12, 0.26)	13; 7 (p = 0.779)
c. Fractures			
Femoral fractures	35/121 (28.9%)	0.35 (0.32, 0.38)	25; 10 (p = 0.829)
Other bone fractures	38/121 (31.4%)	0.33 (0.31, 0.36)	24; 14 (p = 0.563)

Event rates are expressed as n/N (x.x%); n = number of patients with at least one event; N = total number of individuals at risk (excluded if ‘time-to-event’ was not available/missing)

*GI* gastrointestinal, *CI* confidence interval, *NA* not applicable

### Renal complications

Twenty-eight patients experienced at least one renal complication at a median age of 20.75 years old (IQR 15.6–26.3; 6/28 at unknown age). Bladder dysfunction was the most prevalent among renal complications (n = 15). Five patients suffered from acute kidney injury (AKI), which was the primary cause of death in one patient, while three patients developed AKI within the last three months before death -of whom two had established heart failure-. The incidence rate for AKI was 16.6 per 10,000 person-years (Table 2). Other renal complications were chronic renal failure (n = 6) and recurrent urinary tract infections (n = 2). Renal complications were significantly more common among patients who died of cardiopulmonary compared to other causes of death (p < 0.001).

**Table 2:** Incidence rates for severe comorbidities and fractures

Complication or event	Number of observed patients	Incidence rate (per person-years)	95% CI
Acute kidney injury	123	16.56/10,000	2.04–31.07/10,000
Acute myocardial infarction	119	17.26/10,000	2.13–32.39/10,000
Life-threatening arrhythmia	119	48.34/10,000	23.02–73.66/10,000
Severe GI complications	114	56.83/10,000	28.98–84.67/10,000
Hepatic/biliary/pancreatic disorder	114	39.44/10,000	16.13–62.74/10,000
Venous thromboembolism	122	23.52/10,000	6.10–40.95/10,000
Cerebrovascular event	122	23.52/10,000	6.10–40.95/10,000
Any fracture	121	310/10,000	246.69–373.41/10,000

*GI* gastrointestinal, *CI* confidence interval

### Acute cardiac events

Acute myocardial infarction occurred in five patients (4.2%), resulting in death in two of them. Life-threatening arrhythmia (11.8%, n = 14) was successfully managed with cardioversion, electrical or chemical, in nine patients, being fatal for the remaining five patients. The incidence rates are outlined in Table 2.

### Severe GI complications and GI dysfunction

Severe GI complications were seen in 16 patients at a median age of 25.5 years while hepatic, biliary or pancreatic disorder in 11 patients at a median age of 18.5 years (Table 2). These complications constituted the primary cause of death in six patients at a median age of 22.8 years old. These were, in particular, GI infection (n = 2; gastroenteritis and peritonitis), pancreatitis (n = 1), intestinal volvulus (n = 2) and GI hemorrhage (n = 1). In total, seven patients suffered from ileus or volvulus and three patients from GI hemorrhage.

**Fig. 2:**
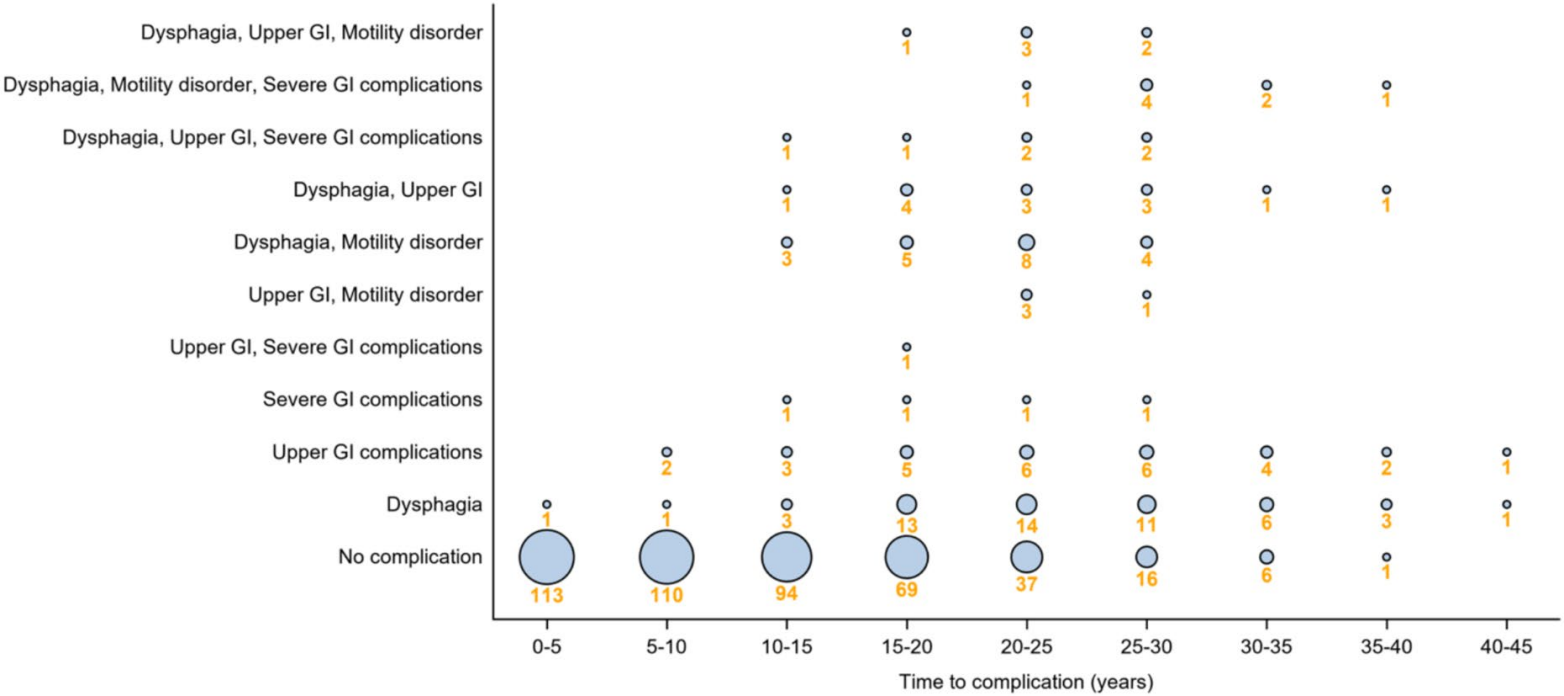
The occurrence of any gastrointestinal (GI) dysfunction either alone or in combination by age of occurrence (N = 114)

Gastrointestinal dysfunction besides mild/moderate constipation or gastrostomy-related complications, occurred in 67.5% of patients (n = 77/114), and was first diagnosed after loss of ambulation in all but three patients. The most common was dysphagia, followed by motility disorder (Figs. 1b and 2). There was no significant correlation between treatment with glucocorticoids and the development of GI dysfunction in general; however, upper GI complications were more common among patients treated with glucocorticoids (HR 95% CI 2.28 (0.88, 5.90), p = 0.088). Dysphagia was less common among patients born after 1990, compared to the earlier birth cohort (Table 1; Supplementary Fig. 1). Of the 9 patients born after 1990 with dysphagia, only two patients were on glucocorticoids at the time they developed dysphagia.

### Skeletal events and bone health

**Fig. 3:**
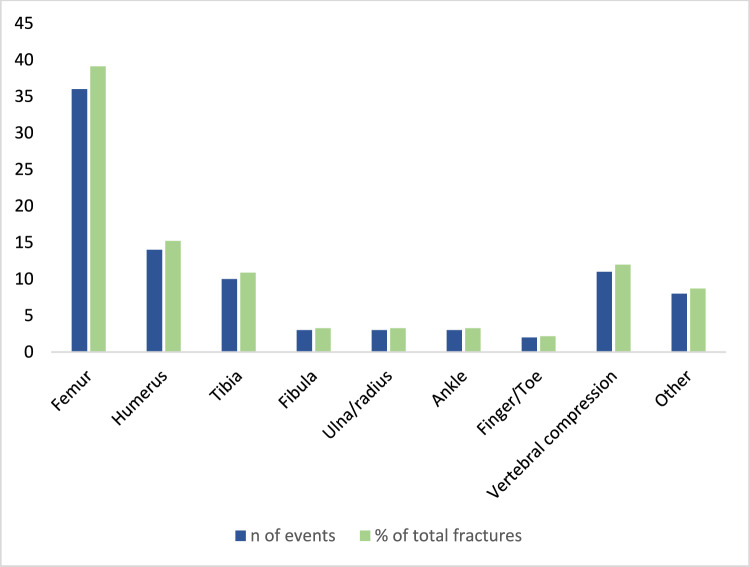
Frequency and localization of fractures (N = 121)

Fifty-eight patients (47.9%, n = 58/121) had sustained at least one fracture, with onset at a median age of 12 years old (IQR 9.5–13.2). The incidence rate of fracture occurrence was 310 per 10,000 person-years (Table 2). Sixteen of these 58 patients (27.6%) were ambulatory when the initial fracture took place. The most common type of fracture was femoral (28.9%, n = 35/121), which was slightly less prevalent compared to all other bone fractures together (33.9%, n = 41/121). The frequency and localization of fractures is shown in Fig. 3.

**Fig. 4:**
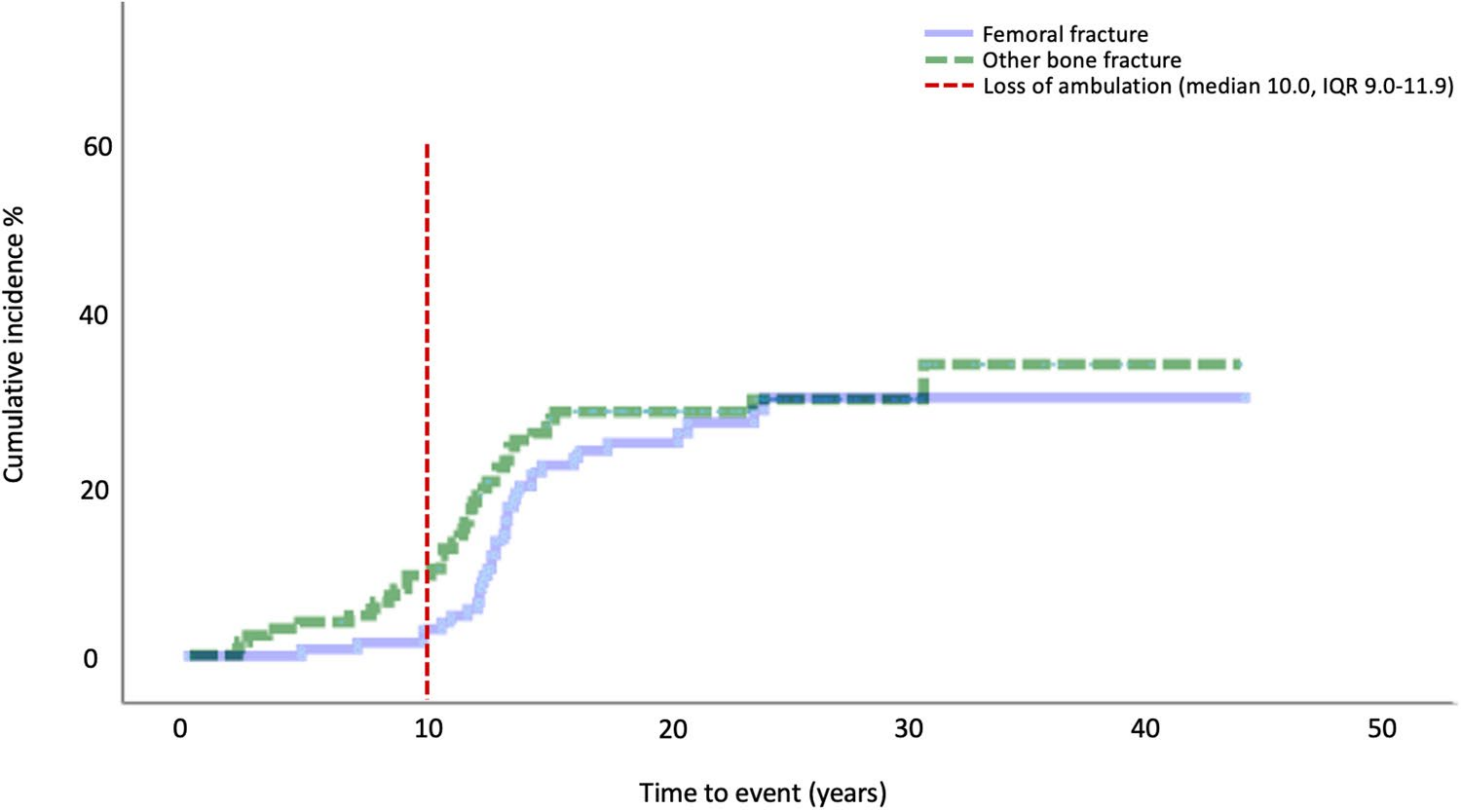
Cumulative incidence of femoral and other bone fractures (N = 121)

Femoral fractures occurred at a slightly older age (median 13.1 years old, IQR 12.1–14.3), compared to other bone fractures (median 11.5 years old, IQR 8.7–13.0), with only 2 of 35 patients being ambulatory at the time of femoral fracture occurrence. The cumulative incidence in relation to loss of ambulation is shown in Fig. 4. Eight patients sustained fractures in adulthood (median 23.7 years old, IQR 20.4–30.3). Approximately 15% of patients (n = 18/121) sustained at least one recurrent fracture. Fracture recurrence was more common among patients on glucocorticoids compared to steroid-naive patients (p = 0.025), while there was no association between glucocorticoids and singular fracture (p = 0.45). Four patients died of post-traumatic pulmonary embolism. Osteoporosis had been diagnosed prior to any fracture in 15.7% of patients (n = 19) at a median age of 15.3 years old (IQR 13.3–16.9).

### Vascular events

In total, vascular events were identified in 13 patients (10.7%) at a median age of 23.5 years (IQR 17.9–28.7). Seven of these patients developed venous thromboembolism, in particular post-traumatic pulmonary embolism (n = 4) and deep venous thrombosis (n = 3). The incidence rate of venous thromboembolism was 23.5 per 10,000 person-years (Table 2).

Cerebrovascular events occurred in 6 patients (4.9%) accounting for an incidence rate of 23.5 per 10,000 person-years (Table 2). Two of these patients were diagnosed with transient ischemic attack at a median age of 36 years. The remaining 4 patients underwent either ischemic (n = 2) or hemorrhagic stroke (n = 1) or both (n = 1; strokes occurred at different time points), all under the age of 25 years. Cardiomyopathy with left ventricular ejection fraction below 45% was evident at the time of the event in 5 of 6 patients, with detectable cardiac thrombus in one of them. None of these 6 patients had previously experienced any acute cardiac event or arrhythmia except for sinus tachycardia. Two patients were on glucocorticoids at the time of the event, while 3 of 6 patients were steroid-naive. One patient was on anticoagulant medication, due to a femoral fracture. This was one of the 2 patients experiencing a transient ischemic attack. Cerebral infarction was the primary cause of death in 2 of these 6 patients.

## Discussion

This population-based study yields new insights into severe, less common comorbidities in DMD. Over 50% of the individuals in our study population sustained at least one severe complication or event, namely life-threatening arrhythmia, post-traumatic pulmonary embolism, cerebrovascular event, acute kidney injury or severe GI complication. We studied the incidence rates of these serious complications and events, illustrating their occurrence across the lifespan of patients with DMD. We found an increased occurrence of life-threatening events or complications during late adolescence and early adulthood, which also contributed to the younger age of patients dying from non-cardiopulmonary causes of death compared to the rest of the study population. The fact that 64% of patients with life-threatening arrhythmias were successfully treated underscores the importance of not only conducting regular rhythm evaluations irrespective of age, but also of prompt rhythm assessment upon the emergence of new cardiovascular symptoms or unexpected clinical deterioration.

Venous thromboembolic events, such as deep vein thrombosis and pulmonary embolism, are rare but severe complications after trauma and lower extremity fracture, carrying substantial morbidity and mortality [10]. Femur fracture in particular is a well-recognized risk factor for pulmonary embolism and fat embolism syndrome in the immediate post-traumatic period [11, 12]. Four patients in our cohort died of post-traumatic pulmonary embolism. The most common type of fracture was femoral, occurring almost exclusively among non-ambulatory patients. Fracture occurrence during adulthood was reported in 6.6% of our study population. The persistent occurrence of low-energy long-bone fractures among adults with DMD has been recently reported, along with a significantly higher risk for those patients on glucocorticoids [13]. Although not associated with singular fracture, the use of glucocorticoids in our study significantly correlated to fracture recurrence. Furthermore, we found that approximately half of our study population had sustained at least one fracture with an overall incidence rate of 310 per 10,000 person-years, in alignment with previous studies in DMD reporting incidence rates between 300 and 890 per 10,000 person-years [13–15]. Our results add to the literature emphasizing the need for regular monitoring of bone health throughout adolescence and adulthood, as well as for the implementation of a validated fracture risk assessment tool. Clinicians should maintain a high index of suspicion for thromboembolic events in DMD patients presenting with sudden onset of symptoms following trauma, even in the absence of overt fractures. Assessment for a hypercoagulable state after injury in these cases is imperative.

Cerebral infarction in DMD has been reported as more prevalent compared to the general population, with 100-times higher incidence than that of ischemic infarction among young male adults [16]. Our findings concur with existing literature showing that cardiomyopathy with low ejection fraction, even in the absence of arrhythmia, constitutes a significant risk factor [16, 17]. Associations between long-term exposure to oral corticosteroids and risk for cerebrovascular events has not been comprehensively investigated, with limited evidence reporting either increased or no risk among adults in general [18, 19]. No association was found between glucocorticoids and stroke occurrence in our study, neither has such an association been demonstrated in other studies in DMD. In clinical practice, careful assessment of the individual risk profile for cardioembolic events is essential to guide appropriate treatment, while equal attention should be directed toward the timely recognition of symptoms, especially among adolescents and young adults with DMD.

Another comorbidity that has been associated with poor outcome especially among adults with DMD, is GI dysmotility and pseudo-obstruction [20, 21]. In our study, the overall incidence of severe GI complications was 56.8 per 10,000 person-years, constituting the primary cause of death in six patients. Impaired GI transport with collection of intestinal air and chronic constipation is considered a major but not singular underlying cause. Our findings suggest that GI infections and hemorrhage also contribute to morbidity and mortality in DMD. Furthermore, aging with DMD and the cardiopulmonary burden that ensues may render these patients non-perceptive to symptoms of impaired GI function and severe constipation, leading to underdiagnosis and inadequate treatment [21, 22]. While treatment with glucocorticoids has been reported to reduce the risk of dysphagia and constipation [23], our study did not find a direct correlation between glucocorticoid use and GI dysfunction. We found a lower frequency of dysphagia among patients born after 1990, potentially linked to the introduction of glucocorticoids. Differences from previous study results may be attributed to different study designs, including the broader definition of GI dysfunction used in our study and variations in how treated and untreated groups were classified. Proactive GI monitoring with routine assessment of nutrition and bowel habits, motility assessment tools and targeted imaging when indicated, is an integral part of the multidisciplinary care and should persist throughout and after the transition to adult care.

The strength of this study was the population-based design and cause-of-death approach allowing us to follow the incidence of serious, less common complications and events across the patients’ lifespan. There were however certain limitations to be acknowledged. This was a retrospective study relying on medical records, thus complete data on comorbidities could not be ensured. Due to a number of missing data, such as fracture patterns and bone health assessments, it was not possible to differentiate between low- versus high-energy fractures or to evaluate the extent of osteoporosis. Confounding factors such as compliance to treatments, polypharmacy, invasive procedures etc. may have been undermeasured and therefore not considered for presentation. The results from the stratified analysis by birth cohort should be interpreted with caution, given the substantially shorter lifespan of patients born in the second cohort, i.e. after 1990.

In conclusion, our study emphasizes the importance of raising awareness in regards to less common, severe comorbidities in DMD, especially in light of the increasing life expectancy [3]. Certain life-threatening events can be prevented or successfully treated, thus reducing the risk for escalated morbidity early in life and premature death. Continuing surveillance after transition to adult care is particularly important to ensure that evolving, potentially life-threatening complications are not overlooked amid the increasing cardiopulmonary burden of disease.

## Supplementary Information

Below is the link to the electronic supplementary material.

Supplementary file1 (DOCX 195 KB)

## Data Availability

The data that support the findings of this study are available from the corresponding author upon reasonable request.

## References

[1] Crisafulli S, Sultana J, Fontana A, Salvo F, Messina S, Trifirò G (2020) Global epidemiology of Duchenne muscular dystrophy: an updated systematic review and meta-analysis. Orphanet J Rare Dis 15(1):141. 10.1186/s13023-020-01430-8 32503598 10.1186/s13023-020-01430-8 PMC7275323

[2] Broomfield J, Hill M, Guglieri M, Crowther M, Abrams K (2021) Life expectancy in Duchenne muscular dystrophy: Reproduced individual patient data meta-analysis. Neurology 97(23):e2304–e2314. 10.1212/WNL.0000000000012910 34645707 10.1212/WNL.0000000000012910 PMC8665435

[3] Wahlgren L, Kroksmark AK, Tulinius M, Sofou K (2022) One in five patients with Duchenne muscular dystrophy dies from other causes than cardiac or respiratory failure. Eur J Epidemiol 37(2):147–156. 10.1007/s10654-021-00819-4 34802091 10.1007/s10654-021-00819-4 PMC8960570

[4] Vaillend C, Aoki Y, Mercuri E, Hendriksen J, Tetorou K, Goyenvalle A, Muntoni F (2025) Duchenne muscular dystrophy: recent insights in brain related comorbidities. Nat Commun 16(1):1298. 10.1038/s41467-025-56644-w 39900900 10.1038/s41467-025-56644-w PMC11790952

[5] Birnkrant DJ, Bushby K, Bann CM, Alman BA, Apkon SD, Blackwell A, Case LE, Cripe L, Hadjiyannakis S, Olson AK, Sheehan DW, Bolen J, Weber DR, Ward LM, DMD Care Considerations Working Group (2018) Diagnosis and management of Duchenne muscular dystrophy, part 2: respiratory, cardiac, bone health, and orthopaedic management. Lancet Neurol 17(4):347–361. 10.1016/S1474-4422(18)30025-5 29395990 10.1016/S1474-4422(18)30025-5 PMC5889091

[6] Mendell JR, Sahenk Z et al (2020) Assessment of systemic delivery of rAAVrh74.MHCK7.micro-dystrophin in children with Duchenne muscular dystrophy: a nonrandomized controlled trial. JAMA Neurol 77(9):1122–1131. 10.1001/jamaneurol.2020.1484 32539076 10.1001/jamaneurol.2020.1484 PMC7296461

[7] Mullard A (2023) FDA approves first gene therapy for Duchenne muscular dystrophy, despite internal objections. Nat Rev Drug Discov 22(8):610. 10.1038/d41573-023-00103-y 37353665 10.1038/d41573-023-00103-y

[8] Chulanova Y, Breier D, Peer D (2025) Delivery of genetic medicines for muscular dystrophies. Cell Rep Med 6(1):101885. 10.1016/j.xcrm.2024.101885 39765231 10.1016/j.xcrm.2024.101885 PMC11866442

[9] Wahlgren L, Kroksmark AK, Lindblad A, Tulinius M, Sofou K (2024) Respiratory comorbidities and treatments in Duchenne muscular dystrophy: impact on life expectancy and causes of death. J Neurol 271(7):4300–4309. 10.1007/s00415-024-12372-7 38630313 10.1007/s00415-024-12372-7 PMC11233294

[10] Coleman JJ, Zarzaur BL, Katona CW, Plummer ZJ, Johnson LS, Fecher A, O’Rear JM, Feliciano DV, Rozycki GS (2015) Factors associated with pulmonary embolism within 72 hours of admission after trauma: a multicenter study. J Am Coll Surg 220(4):731–736. 10.1016/j.jamcollsurg.2014.12.032 25724603 10.1016/j.jamcollsurg.2014.12.032

[11] Feder D, Koch ME, Palmieri B, Fonseca FLA, Carvalho AAS (2017) Fat embolism after fractures in Duchenne muscular dystrophy: an underdiagnosed complication? A systematic review. Ther Clin Risk Manag 13:1357–1361. 10.2147/TCRM.S143317 29066903 10.2147/TCRM.S143317 PMC5644602

[12] Kim YJ, Choi DH, Ahn S, Sohn CH, Seo DW, Kim WY (2016) Timing of pulmonary embolisms in femur fracture patients: Incidence and outcomes. J Trauma Acute Care Surg 80(6):952–956. 10.1097/TA.0000000000001014 26891161 10.1097/TA.0000000000001014

[13] Langlands G, McKechnie J, Farrugia ME, Wong SC (2025) Incidence of radiologically confirmed fractures in adults with Duchenne muscular dystrophy. Muscle Nerve 71(4):558–563. 10.1002/mus.28355 39844691 10.1002/mus.28355

[14] Joseph S, Wang C, Bushby K, Guglieri M, Horrocks I, Straub V, Ahmed SF, Wong SC, UK NorthStar Clinical Network (2019) Fractures and linear growth in a nationwide cohort of boys with Duchenne muscular dystrophy with and without glucocorticoid treatment: results from the UK NorthStar database. JAMA Neurol 76(6):701–709. 10.1001/jamaneurol.2019.0242 30855644 10.1001/jamaneurol.2019.0242 PMC6563545

[15] Liaw J, Billich N, Carroll K, Adams J, Ryan MM, Yiu EM, Zacharin M, Simm P, Davidson ZE (2023) Fracture risk and impact in boys with Duchenne muscular dystrophy: a retrospective cohort study. Muscle Nerve 67(6):489–496. 10.1002/mus.27762 36478256 10.1002/mus.27762

[16] Nozaki F, Kusunoki T, Kumada T, Shibata M, Fujii T (2019) Risk factors for cerebral infarction in Duchenne muscular dystrophy: review with our 2 cases. J Stroke Cerebrovasc Dis 28(9):2453–2458. 10.1016/j.jstrokecerebrovasdis.2019.06.023 31311695 10.1016/j.jstrokecerebrovasdis.2019.06.023

[17] Winterholler M, Holländer C, Kerling F, Weber I, Dittrich S, Türk M, Schröder R (2016) Stroke in Duchenne muscular dystrophy: a retrospective longitudinal study in 54 patients. Stroke 47(8):2123–2126. 10.1161/STROKEAHA.116.013678 27354222 10.1161/STROKEAHA.116.013678

[18] Jang YH, Choi EY, Lee H, Woo J, Park S, Noh Y, Jeon JY, Yoo EY, Shin JY, Lee YW (2024) Long-term use of oral corticosteroids and safety outcomes for patients with atopic dermatitis. JAMA Netw Open 7(7):e2423563. 10.1001/jamanetworkopen.2024.23563 39028668 10.1001/jamanetworkopen.2024.23563 PMC11259904

[19] Souverein PC, Berard A, Van Staa TP, Cooper C, Egberts AC, Leufkens HG, Walker BR (2004) Use of oral glucocorticoids and risk of cardiovascular and cerebrovascular disease in a population based case-control study. Heart 90(8):859–865. 10.1136/hrt.2003.020180 15253953 10.1136/hrt.2003.020180 PMC1768386

[20] Blokhuis AM, Tytgat K, Groothuis JT, Houwen-van OS (2024) Severe gastrointestinal problems in Duchenne muscular dystrophy: A case series. Neuromuscul Disord 40:31–37. 10.1016/j.nmd.2024.05.006 38823288 10.1016/j.nmd.2024.05.006

[21] Lo Cascio CM, Goetze O, Latshang TD, Bluemel S, Frauenfelder T, Bloch KE (2016) Gastrointestinal dysfunction in patients with Duchenne muscular dystrophy. PLoS ONE 11(10):e0163779. 10.1371/journal.pone.0163779 27736891 10.1371/journal.pone.0163779 PMC5063332

[22] Kraus D, Wong BL, Horn PS, Kaul A (2016) Constipation in Duchenne muscular dystrophy: prevalence, diagnosis, and treatment. J Pediatr 171:183–188. 10.1016/j.jpeds.2015.12.046 26831528 10.1016/j.jpeds.2015.12.046

[23] Schiava M, Lofra RM, Bourke JP, James MK, Díaz-Manera J, Elseed MA, Michel-Sodhi J, Moat D, Mccallum M, Mayhew A, Ghimenton E, Díaz CFB, Malinova M, Wong K, Richardson M, Tasca G, Grover E, Robinson EJ, Tanner S, Eglon G, Behar L, Eagle M, Turner C, Verdú-Díaz J, Heslop E, Straub V, Bettolo CM, Guglieri M (2024) Disease-associated comorbidities, medication records and anthropometric measures in adults with Duchenne muscular dystrophy. Neuromuscul Disord 41:8–19. 10.1016/j.nmd.2024.05.007 38865917 10.1016/j.nmd.2024.05.007

