# Supplementary material for: A population-based study of severe, less common comorbidities in Duchenne muscular dystrophy
Source: J Neurol. 2025 Aug 28;272(9):596. doi: 10.1007/s00415-025-13323-6 (PMC12394381; doi:10.1007/s00415-025-13323-6)
Supplement: Supplementary file 1 — Supplementary file1 (DOCX 195 KB) [file 415_2025_13323_MOESM1_ESM.docx]

**Supplementary Figure 1:** Cumulative incidence of dysphagia (n=50; N=109 observed). Patients are grouped in two birth cohorts; 1970-1989 and 1990-2009.

**
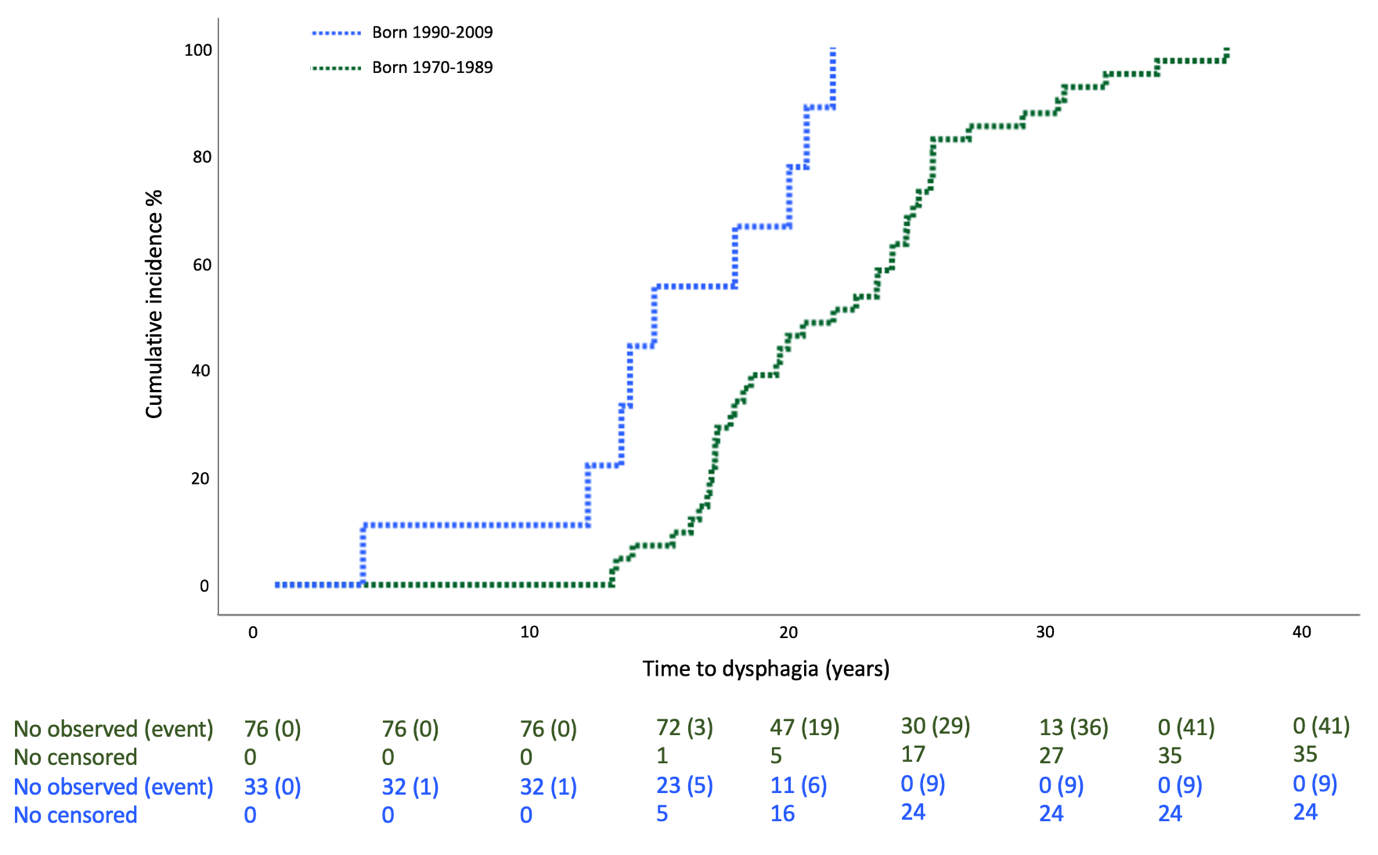
**
